# High-Throughput Sequencing Analysis of the Composition and Diversity of the Bacterial Community in *Cinnamomum camphora* Soil

**DOI:** 10.3390/microorganisms10010072

**Published:** 2021-12-30

**Authors:** Deqiang Chen, Weihong Sun, Shuang Xiang, Shuangquan Zou

**Affiliations:** 1 College of Forestry, Fujian Agriculture and Forestry University, Fuzhou 350002, China; deqiang@fafu.edu.cn (D.C.); swhjaponica@sina.com (W.S.); Xiang@fafu.edu.cn (S.X.); 2 Fujian Colleges and Universities Engineering Research Institute of Conservation and Utilization of Natural Bioresources, College of Forestry, Fujian Agriculture and Forestry University, Fuzhou 350002, China; 3 Key Laboratory of National Forestry and Grassland Administration for Orchid Conservation and Utilization at Colleage of Landscape Architecture, College of Landscape Architecture, Fujian Agriculture and Forestry University, Fuzhou 350002, China

**Keywords:** bacterial communities, *Cinnamomum camphora*, root-associated microbiomes, soil diversity

## Abstract

Soil bacterial communities and root-associated microbiomes play important roles in the nutrient absorption and healthy growth of host plants. *Cinnamomum camphora* is an important timber and special economic forest tree species in Fujian Province. In this study, the high-throughput sequencing technique was used to analyze the composition, diversity, and function of the bacterial communities present in the soil from different samples and slope positions of *C. camphora*. The results of this analysis demonstrated that the related bacterial communities in *C. camphora* soil were mainly clustered based on sample type. Bacterial alpha diversity in the rhizosphere and bulk soil of *C. camphora* growing downhill was higher than that of *C. camphora* growing uphill. At the phylum level, Bacteroidetes, Proteobacteria, Chloroflexi, and Gemmatimonadetes were positively correlated with pH, available phosphorus, total phosphorus, available potassium, and total potassium, while Acidobacteria and Verrucomicrobia were negatively correlated with alkaline-hydrolyzable nitrogen. These results show that there were remarkable differences in the composition, diversity, and function of related bacterial communities between different sample types of *C. camphora* soil. The slope position had a marked effect on the bacterial communities in the rhizosphere and bulk soil, while the root endosphere remained unaffected.

## 1. Introduction

The microbial community present in soil influences the ecological function of the soil as well as the health of the host plant [1]. There are numerous microorganisms living in the rhizosphere and root endosphere of plants. These root-associated microorganisms play critical roles in plant growth and development, nutrient absorption, and ecological functions [2,3,4,5]. Therefore, it is important to understand the composition and diversity of root-associated microbial communities.

Previous studies reported that the rhizosphere microbiota was evidently different from that of the bulk soil because the root exudates changed the nutrient environment of the rhizosphere [6,7]. Plants can select specific microbial groups from the soil. The number of microorganisms per gram of rhizosphere soil is 10–1000 times that of the bulk soil [8]. In the rhizosphere microbiome of *Avena fatua*, 1917 bacterial groups were discovered, of which 147 had a remarkably greater relative abundance than those in the bulk soil [9]. On comparing the findings of various studies, different species of plants were found to enrich different bacterial communities in the rhizosphere. For example, the rhizosphere bacteria of plants belonging to *Quercus* species mainly include Acidobacteria, Actinobacteria, and Proteobacteria [10]. In the rhizosphere of *Beta vulgaris*, the relative abundance of Acidobacteria is lower than that of Firmicutes [11]. When investigating the root endosphere, many studies have reported that the alpha diversity of bacterial communities in this area is further reduced. Bulgarelli and Lundberg’s research on the root microbes of *Arabidopsis thaliana* revealed that the bacterial richness in the root endosphere was almost half lower than that in the soil [12,13].

The plant rhizosphere is a dynamic environment, and its microbial community can be influenced by various conditions. Soil type is one of the most critical factors in determining the structure of the rhizosphere microbial community [14,15]. Normander and Prosser found that the microbial community in the rhizosphere of *Cucumis sativus* is primarily derived from the soil environment rather than its seeds [16]. Bulgarelli et al. performed pyrosequencing and comparison of 16S rRNA genes of the rhizosphere and root endosphere microorganisms from more than 600 *A. thaliana* plants from different soils and genetic backgrounds. They found that the diversity of rhizosphere bacterial communities was largely influenced by soil type and scarcely by plant genotype [12]. In addition, unique ecological niches may shape the structure of the plant microbiome through the interactions of plant species, plant chemical properties (such as C/N ratio, cellulose, and lignin contents), soil properties, and many other factors. Therefore, different plant locations may have different microbial populations [17,18]. It is reported that the slope position is an important topographic factor that controls the microenvironment heterogeneity by affecting plant temperature, light, physical and chemical properties of the soil, and water level [19,20]. Although slope position is not a direct ecological factor determining the survival of microorganisms, it can affect the distribution of microorganisms by controlling the spatial-temporal distribution of a series of ecological factors and their combinations [21]. Therefore, the soil type and slope position of plants are closely related to the microbial community. Further in-depth research is needed to systematically study the relationship between the two.

*Cinnamomum camphora* belongs to the family Lauraceae. It is mainly distributed in tropical and subtropical Asia [22], and it is an important timber and special economic forest tree species in Fujian Province [23]. *C. camphora* can not only be used in wood processing and interior decoration, but it also has great medicinal and economic value [24]. At present, existing research on *C. camphora* mainly focuses on the composition of its essential oil, seedling technology, and secondary metabolites. A few other scholars have described and compared endophytic bacterial communities in *C. camphora* leaves during different seasons [25] as well as analyzed the diversity and metabolism of endophytic bacterial communities in its leaves [26]. The diversity of soil bacterial communities in the rhizosphere of *C. camphora* has only been studied in recent literature [27]. However, the related bacterial communities in different soil samples and from different slope positions of *C. camphora* have not yet been studied; these topics need to be discussed in further detail.

In this study, high-throughput sequencing technology was used to analyze the bacterial diversity and community composition in the rhizosphere, bulk soil, and root endosphere of *C. camphora*. In addition, the influence of soil chemical properties and slope position on the bacterial community, as well as the changes in the predictive function of the bacterial community, were analyzed. This study aimed to provide some insight into the research on the bulk soil and root-associated bacterial communities of *C. camphora*. Furthermore, it offers a scientific basis for the sustainable growth of *C. camphora*.

## 2. Materials and Methods

### 2.1. Experimental Procedure

Six test plots were selected from a 5-year-old unharvested *C. camphora* forest located in the Banlin State-owned Forest Farm in Anxi, Fujian Province, with three plots on the upslope and three on the downslope. The plots are located at an altitude of between 718 and 823 m and at 24°56′39″–24°57’3″ N, 117°58′46″–118°0′20″ E. The region has a subtropical monsoon climate, with hot and rainy climate in summer and mild and humid climate in winter, an average annual temperature of 18–19 °C, a maximum temperature of 39 °C, minimum temperature of 0 °C, and an annual average rainfall of 1600–1800 mm. The elevation difference between the up- and downslope areas is approximately 100 m. Each test plot measured 10 × 10 m and contained 60 *C. camphora* plants. The average tree height, ground diameter, crown width, and total biomass of the three sample plots on the uphill slope were 291.5–372.8 cm, 5.5–6.2 mm, 148.2–190.8 cm, and 2635.9–4544.8 kg, respectively. The average tree height, ground diameter, crown width, and total biomass of the three plots on the downhill slope were 371.1–423.3 cm, 5.9–7.5 mm, 145.4–222.3 cm, and 6331.4–7947.7 kg, respectively.

Sampling was conducted in early June 2020, during the summer shoot growth period of *C. camphora*. In each test site, a 0–20 cm soil layer was selected from five points between the rows of *C. camphora*, according to the five-point method, and combined into a soil sample. Approximately 500 g of soil was selected using the quartering method, stored in a sealed bag, and returned to the laboratory for air-drying; the physical and chemical properties of this sample were then determined. The chemical properties of the soil sample such as pH, alkaline-hydrolyzable nitrogen (AN), total phosphorus (TP), available phosphorus (AP), available potassium (AK), and total potassium (TK) were tested. In addition, the root endosphere, rhizosphere, and bulk soil samples of the 0–20 cm soil layer of *C. camphora* were collected, and the root samples were immediately pretreated to minimize changes in the root endophytic microbial community. The rhizosphere soil was then placed in a sterilized 5 mL centrifuge tube using a sterilized spoon and forceps. Three trees were selected from each plot; a total of 54 samples were collected, and they were brought back to the laboratory and placed in a refrigerator at −80 °C for the extraction of microbiological DNA and high-throughput bacterial sequencing.

### 2.2. Sample Preparation and DNA Extraction

The rhizosphere and root endosphere samples were collected using the methods described by Xiao et al. [28]. Root samples were placed in a 50 mL sterile centrifuge tube with 25 mL of phosphate buffer and were shaken on a vortex shaker for 15 s at the maximum speed of 3200 rpm. The shaken suspension was filtered through a 100 μm sterile nylon mesh into a new centrifuge tube and centrifuged at 3200× *g* for 15 min. The supernatant was discarded, and the remaining precipitate was the rhizosphere soil, which was stored in the refrigerator at −80 °C.

The total DNA of the sample was extracted using the TGuide S96 Magnetic Soil DNA Kit (Tiangen Biotech (Beijing) Co., Ltd., Beijing, China) according to manufacturer instructions. The DNA concentration of the samples was measured with the Qubit dsDNA HS Assay Kit and Qubit 4.0 Fluorometer (Invitrogen, Thermo Fisher Scientific, Waltham, MA, USA).

### 2.3. 16S rRNA Gene Amplification and Sequencing

Primers 338F (5′-ACTCCTACGGGAGGCAGCA-3′) and 806R (5′-GGACTACHVGGGTWTCTAAT-3′) were used to amplify the V3 + V4 variable region of the 16S rRNA gene of all DNA samples [29]. The target area for polymerase chain reaction (PCR) (10 μL) included 5–50 ng genomic DNA, two primers Vn F and Vn R of 0.3 μL each, 5 μL of the KOD FX Neo buffer, 2 μL of deoxynucleoside triphosphate (2 mM each), 0.2 μL of the KOD FX Neo buffer, and double-distilled water supplemented to 10 μL. The PCR conditions were as follows: denaturation at 95 °C for 5 min and amplification 25 times (at 95 °C for 30 s, 50 °C for 30 s, and 72 °C for 40 s), followed by a final extension at 72 °C for 7 min. The total of PCR amplicons was purified with Agencourt AMPure XP Beads (Beckman Coulter, Indianapolis, IN) and quantified using the Qubit dsDNA HS Assay Kit and Qubit 4.0 Fluorometer (Invitrogen, Thermo Fisher Scientific, Waltham, MA, USA). After the individual quantification step, amplicons were pooled in equal amounts. For the constructed library, use Illumina novaseq 6000 for sequencing.

### 2.4. Sequence Analysis

The obtained original sequences were spliced and sequenced using the FLASH v1.2.11 software [30], and the Trimmomatic v0.33 software [31] was then used to filter the spliced sequences to obtain high-quality sequences. The chimeras were removed using the UCHIME v8.1 software [32] to obtain high-quality tag sequences. The sequences were clustered using the USEARCH v10.0 software [33], with a similarity level of 97%. Operational taxonomic units (OTU) were filtered with a threshold of 0.005% for all sequences that were sequenced [34]. Representative sequences from OTUs were species-annotated using the RDP classifier v2.2 [35] (80% confidence interval) based on the Silva database (Release 128, http://www.arb-silva.de, accessed on 10 August 2021) [36]. Removal of the annotation resulted in chloroplast and mitochondrial OTUs or OTUs with only one sequence. Considering the difference in the sequencing depth of different samples, we screened 1996 sequences and flattened them for subsequent analysis. The original sequences obtained by sequencing have been uploaded to the NCBI SRA database, and the BioProject number is PRJNA779380.

### 2.5. Statistical Analyses

All statistical analyses were performed using the R v4.1.1 software. The permutational multivariate analysis of variance (PERMANOVA) analysis was performed with the vegan package, and all samples were tested for a significant difference in beta diversity. The Bray–Curtis difference was used to study the beta diversity of the bacterial community structure. The unconstrained principal coordinate analysis (PCoA) was used to further visualize the bacterial community structure. ANOVA was used to study the differences in the relative abundance of species between sample types (*p*  <  0.05). The Edger software package was used for statistical analysis of the richly differentiated OTUs [37], and they were visualized using the ggplot2 package.

PICRUSt2 was applied to perform species annotation on feature sequences based on a reference phylogenetic tree. Potential functions and functional genes in samples were predicted based on the Kyoto Encyclopedia of Genes and Genomes (KEGG) database [38]. The significance of the difference in function abundance between samples was evaluated by G-TEST (number of annotated functional genes > 20) and Fisher (number of annotated functional genes < 20) in STAMP (*p*  <  0.05).

## 3. Results

### 3.1. Distinct Bacterial Communities in Root Endosphere and Rhizosphere

**Figure 1:**
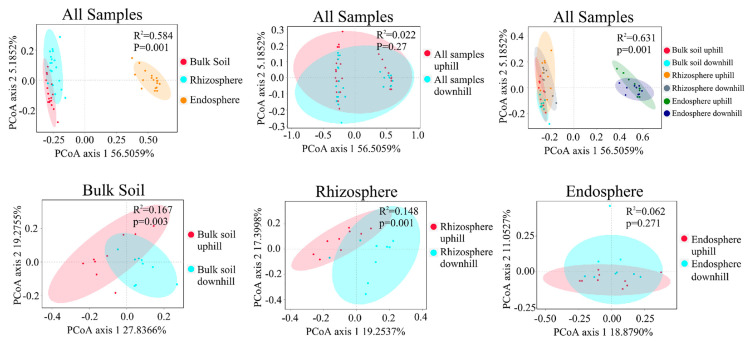
PCoA plot showing the variations in bacterial communities based on the Bray–Curtis distance (PERMANOVA was used to test for significant differences). Ellipses indicate 95% confidence intervals for each sample type.

**Figure 2:**
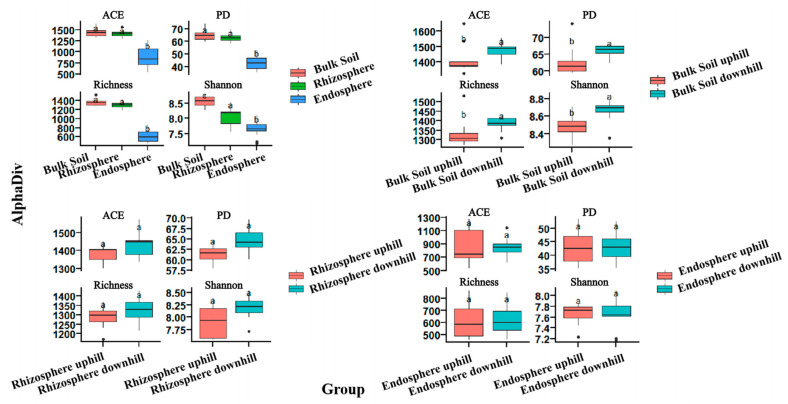
Alpha diversity indices of bacterial communities in root endosphere, rhizosphere, and bulk soil samples, including estimates such as abundance-based coverage estimator (ACE), Faith’s phylogenetic diversity (PD), richness, and Shannon index. Different letters represent significance at *p* < 0.05.

Unconstrained PCoA showed that soil type is the main clustering factor for bacterial communities, followed by location. The rhizosphere and bulk soil samples were closely clustered together and were away from the root endosphere samples. There were significant differences (*p* < 0.05) in the rhizosphere and bulk soil bacterial community composition between the up- and downslope areas. In contrast, there was no significant difference between the root endosphere samples from the up- and downslope areas (Figure 1). The measurement of diversity within the sample (alpha diversity) revealed the diversity gradient from the endosphere to the bulk soil. The alpha diversity of the bulk soil samples was the highest, while that of the root endosphere samples was the lowest, according to estimates such as abundance-based coverage estimator (ACE), Faith’s phylogenetic diversity (PD), richness, and Shannon index. In addition, the alpha diversity of the downslope of the rhizosphere soil and bulk soil samples was higher than that of the upslope samples. Similarly, there was no significant difference in alpha diversity between the up- and downslope samples of the root endosphere (Figure 2). The difference in the alpha diversity index among all sample types was statistically significant.

### 3.2. OTUs in Root Endosphere and Rhizosphere

**Figure 3:**
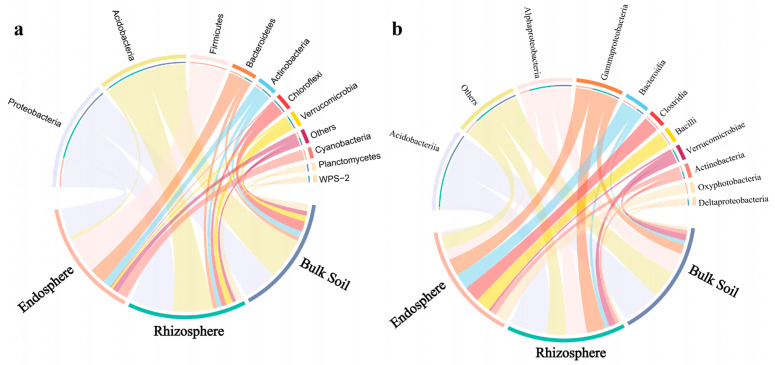
Relative abundance of bacteria at phylum (**a**) and class levels (**b**) based on all samples in each sample type.

**Figure 4:**
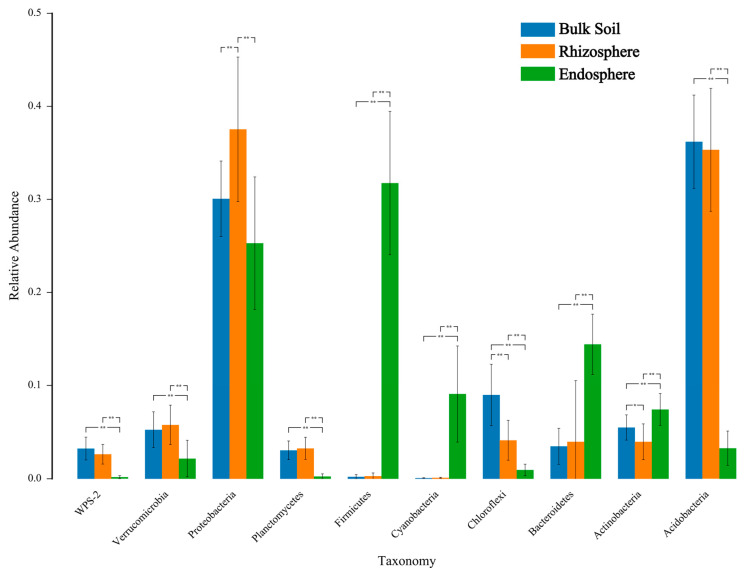
The *X*-axis represents species (the top 10 species with the lowest *p* values are shown); *Y*-axis represents the relative richness of species; columns with different colors represent samples; and * on the columns indicates that the difference is significant (*p* < 0.05), ** means the difference is very significant (*p* < 0.01).

There were significant differences in the relative abundance of samples at different classification levels. At the phylum level, the relative abundance of Proteobacteria in the rhizosphere of *C. camphora* soil accounted for the highest proportion, reaching 37.4%. The bulk soil and rhizosphere of *C. camphora* had a significantly greater proportion of Acidobacteria than the root endosphere. In addition, the proportion of Chloroflexi and Verrucomicrobia in the root endosphere was relatively low. In contrast, the proportion of Firmicutes in the root endosphere was the highest, and Firmicutes were mostly depleted in the bulk soil and rhizosphere. Furthermore, the root endosphere had a significantly greater proportion of Bacteroidetes, Actinobacteria, and Cyanobacteria compared to the bulk soil and rhizosphere (Figure 3a, Table 1). Taxonomic analysis at the class level shows that the enrichment of the Acidobacteria community in the bulk soil and rhizosphere was mainly influenced by a subset of classes, which were predominantly Acidobacteriia and Alphaproteobacteria (Figure 3b). In addition, ANOVA was performed to compare each phylum among the root endosphere, rhizosphere, and bulk soil. The relative abundance of Proteobacteria and Acidobacteria in the rhizosphere and bulk soil was higher than that in the root endosphere. The most abundant bacteria in the root endosphere were Firmicutes, which is consistent with the above results (Figure 4).

**Figure 5:**
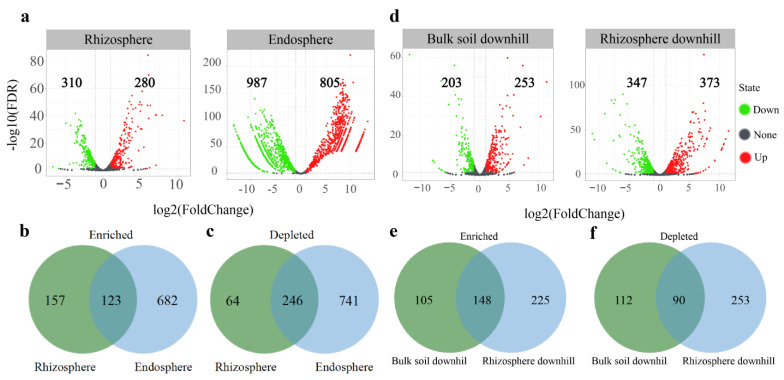
Enriched and depleted OTUs in the root endosphere, rhizosphere, downhill bulk soil, and downhill rhizosphere. (**a**) Bulk soil was used as the control for differential abundance analysis. (**b**) Numbers of differentially enriched OTUs between root endosphere and rhizosphere. (**c**) Numbers of differentially depleted OTUs between endosphere and rhizosphere. (**d**) Uphill bulk soil and rhizosphere were used as controls for differential abundance analysis. (**e**) Numbers of differentially enriched OTUs between downhill bulk soil and rhizosphere. (**f**) Numbers of differentially depleted OTUs between downhill bulk soil and rhizosphere. Each point represents an individual OTU, and the position along the *X*-axis represents the abundance fold change compared to bulk soil. The *Y*-axis is -Log 10 (FDR) obtained by correcting the *p*-value of the significant difference. The closer the point is to the top of the graph, the more significant the difference is.

Considering the OTU count of the bulk soil as the control, the rhizosphere and root endosphere were significantly enriched in 280 and 805 OTUs, respectively (Figure 5a). There were some overlaps in the differentially enriched and depleted OTUs in the root endosphere and rhizosphere. We divided these enriched OTUs into three subcommunities. The first subcommunity was classified as fully enriched rhizosphere OTUs (157 OTUs) and was defined as bacteria significantly enriched in the rhizosphere sample, distinguishing this sample type from the bulk soil. The second subcommunity was designated as fully enriched root endosphere OTUs (682 OTUs) if bacteria were significantly enriched in the root endosphere sample, distinguishing this sample type from the bulk soil. The third subcommunity designated OTUs co-enriched in the root endosphere and rhizosphere (123 OTUs), as defined by OTUs enriched in root endosphere and rhizosphere samples, distinguishing these samples from the bulk soil (Figure 5b).

The rhizosphere and root endosphere had a greater effect on excluding microbes than the bulk soil; 310 OTUs were significantly depleted compared to those in the bulk soil. Several OTUs were reduced in the root endosphere (987 OTUs). Most OTUs that decreased in the rhizosphere also decreased in the root endosphere communities (Figure 5c).

We analyzed the differential abundance of the upslope and downslope rhizosphere and bulk soil samples, respectively. Taking the OTU count of uphill bulk soil as the control, the downhill bulk soil was significantly enriched in 253 OTUs and depleted in 203 OTUs. Taking the OTU count of the uphill rhizosphere as the control, the downhill rhizosphere was significantly enriched in 373 OTUs and depleted in 343 OTUs (Figure 5d). Downhill bulk soil and rhizosphere co-enriched 148 OTUs and co-depleted 90 OTUs (Figure 5e,f). It was apparent that the enrichment of OTUs was significantly higher in the downhill samples compared to depleted OTUs in the rhizosphere and bulk soil.

### 3.3. Soil Property and Its Influence on Bacterial Community

In order to study the changes in soil chemistry, we measured soil pH, AN, TP, AP, TK, and AK. The soil pH values of all six test plots of measurements were similar. The AP, TP, AK, and TK contents of the three downslope plots (b1, b2, and b3) were significantly higher than those of the three upslope plots (a1, a2, and a3) (Table S1).

**Figure 6:**
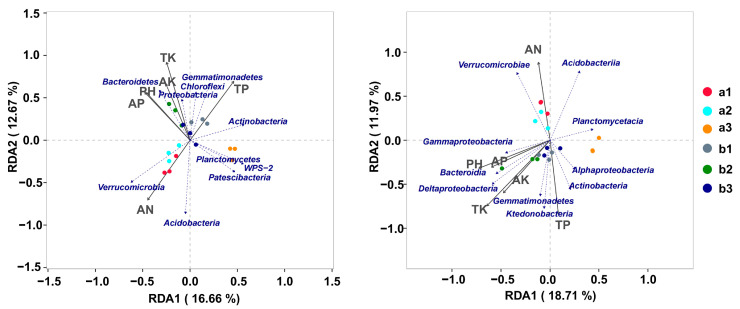
Ordination plots using redundancy analysis (RDA) were used to demonstrate the relationship between bacterial communities and soil properties. The arrows indicate the lengths and angles between explanatory and response variables and reflect their correlations. Different samples are marked with different colors. a1, a2, and a3 represent the bulk soil of the three upslope sample plots, respectively, and b1, b2, and b3 represent the bulk soil of the three downslope sample plots, respectively.

At the phylum level, redundancy analysis (RDA) based on the Bray–Curtis distance revealed that PH, AN, AP, TP, AK, and TK have a significant impact on bacterial communities. Variations in bacterial species generated a strong response to the chemical properties of soil. At the phylum level, dominant bacteria such as Bacteroidetes, Proteobacteria, Chloroflexi, and Gemmatimonadetes exhibited a positive relationship with PH, AP, TP, AK, and TK but a negative relationship with AN. In contrast, Acidobacteria and Verrucomicrobia showed a positive relationship with AN (Figure 6).

Similarly, at the class level, PH, AN, AP, TP, AK, and TK also have a significant impact on bacterial communities. The dominant bacteria such as Gammaproteobacteria, Bacteroidia, and Deltaproteobacteria exhibited a positive relationship with PH, AP, AK, and TK. Among them, Deltaproteobacteria has the greatest correlation. Moreover, Gemmatimonadetes, Ktedonobacteria, Actinobacteria, and Alphaproteobacteria are positively correlated with TP, and Ktedonobacteria has the greatest correlation. In contrast, Verrucomicrobiae and Acidobacteriia showed a positive relationship with AN (Figure 6).

### 3.4. Changes in Predictive Functions of Bacterial Communities

**Figure 7:**
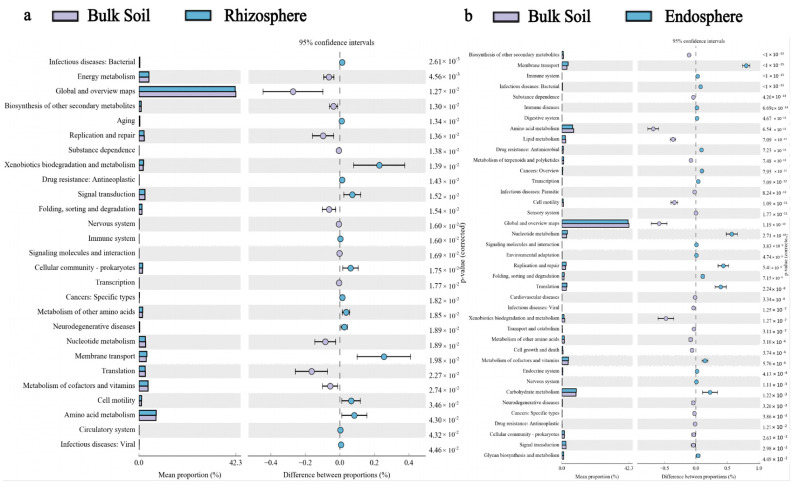
In the second level of the Kyoto Encyclopedia of Genes and Genomes metabolic pathways, with the bulk soil as the control, the differential bacterial functions in the rhizosphere (**a**) and the root endosphere (**b**) were enriched (*p* < 0.05).

The PICRUSt2 analysis based on genome data from the Kyoto Encyclopedia of Genes and Genomes (KEGG) database reveals the functional pathways of the differential expression of bacterial communities in the root endosphere, rhizosphere, and bulk soil of all samples (Figure 7). The functional abundance of xenobiotic biodegradation and metabolism, signal transduction, cellular community-prokaryotes, membrane transport, cell motility, and amino acid metabolism pathways in the rhizosphere soil was higher than those in the bulk soil, and the difference was greater (Figure 7a). Amino acid metabolism and xenobiotic biodegradation and metabolism belong to the metabolism pathway, membrane transport and signal transduction belong to the environmental information processing pathway, and cellular community-prokaryotes and cell motility belong to the cellular process pathway (Table S2). However, the root endosphere was enriched during carbohydrate metabolism, metabolism of cofactors and vitamins, nucleotide metabolism, translation, membrane transport, and replication and repair pathways, and translation; replication and repair belong to the genetic information processing pathway (Figure 7b, Table S2).

## 4. Discussion

### 4.1. Community Composition of C. camphora Root-Associated Bacterial Microbiome

As a general rule of plant root microbial colonization, the alpha diversity index of bacterial communities decreases from the bulk soil to the root endosphere [39]. The alpha diversity gradually decreases from the bulk soil to the root endosphere, indicating that only a part of the bacterial population can maintain a symbiotic association in the root tissue, which becomes the dominant flora after filtration (Figure 2). This means that the root filtration effect is gradually enhanced [40] and the root endosphere has a stronger filtering effect than the rhizosphere.

The slope position is an important topographic factor, and there is a significant difference in species diversity with the change in slope position [21]. Our results found that the alpha diversity of the downslope rhizosphere and bulk soil samples is higher than that of the upslope samples (Figure 2). We suggest that the alpha diversity decreases as the altitude increases. However, the alpha diversity within the root endosphere remains unaffected, which indicates the unique selection effect of the root endosphere.

The enrichment and depletion of certain bacteria in the roots of *C. camphora* show that the bacterial colonization of the *C. camphora* roots is not a passive process. Some bacteria can better occupy the colonization niche of the roots, and plants can select a suitable bacterial community [6]. In our study, Proteobacteria and Firmicutes were significantly enriched, while Acidobacteria and Planctomycetes were significantly depleted in the *C. camphora* root endosphere (Figure 3a, Table 1). Our results are similar to those obtained with rice [41] and *Arabidopsis* [42], showing that at the phylum level, the plant endosphere may select the same or similar dominant bacteria. In addition, the enrichment of Acidobacteria in the rhizosphere samples of *C. camphora* is mainly driven by the enrichment of Acidobacteriia and Alphaproteobacteria at the class level, which is consistent with their rapid growth characteristics. These bacteria can adapt to and use the carbon source in the rhizosphere environment and seize the ecological niche so that their population rapidly increases [43].

By analyzing the difference between the OTU counts in the rhizosphere and root endosphere using the bulk soil as the control, we observed that the rhizosphere was significantly enriched in a part of the bacterial OTUs (Figure 5). In addition, among the OTUs enriched in the rhizosphere, most OTUs were enriched in the root endosphere (Figure 5b). The taxa that settle in the root endosphere may be attracted by elements produced by the root system itself. We found that the rhizosphere can not only be enriched in OTUs but also uniquely enriches a subset of OTUs. This indicates that the rhizosphere is a special niche for certain taxa. In contrast, most of the OTUs depleted in the rhizosphere were also depleted in the root endosphere (Figure 5c). This indicates that environmental colonization is likely to be dominated by the rhizosphere, which may restrict certain bacteria from entering the root endosphere; thus, they play an important gating role [6].

### 4.2. Relationship between Bacterial Community and Soil Chemical Properties

In addition to sunlight, plants absorb the mineral nutrients necessary for their metabolism and growth from the soil [44]. The structure of the microbial community in the soil influences the changes in nutrients and absorption of these nutrients by the plants. The microorganisms present in soil play a key role in the circulation of soil nutrients and are a representation of the quality of the soil and the stability of the ecosystem; as a result, they can affect the circulation and physicochemical characteristics of soil nutrients [45]. The RDA of the bacterial community (at the phylum level) demonstrated that Bacteroidetes, Proteobacteria, Chloroflexi, and Gemmatimonadetes were beneficial in increasing the AP, PH, AK, TK, and TP content in the soil, with Bacteroidetes and Gemmatimonadetes producing remarkable effects. In contrast, Acidobacteria and Verrucomicrobia were found to be beneficial in increasing the AN content. At the class level, Gammaproteobacteria, Bacteroidia, and Deltaproteobacteria were beneficial in increasing the PH, AP, AK, and TK content in the soil, with Deltaproteobacteria producing remarkable effects. Moreover, Gemmatimonadetes, Ktedonobacteria, Actinobacteria, and Alphaproteobacteria were beneficial in increasing the TP content in the soil. In contrast, Verrucomicrobiae and Acidobacteriia were found to be beneficial in increasing the AN content.

Bacteroidetes are responsible for the degradation of complex organic matter in the biosphere, especially in the form of polysaccharides and proteins, and play a beneficial role in the degradation of organic matter [46]. Similarly, most Proteobacteria play an important role in the decomposition and recycling of organic matter [47]. Chloroflexi can generate energy through photosynthesis [48], and Gemmatimonadetes can convert various sugar molecules into vitamins [49]. At the class level, Deltaproteobacteria are globally distributed, metabolically and phylogenetically diverse bacteria with numerous cultured representatives and play essential roles in the global carbon, sulfur, and nutrient cycling [50]. Species in the class Ktedonobacteria are very diverse in habitats. Moreover, the class Ktedonobacteria developed a spore-forming morphology to survive nutrient depletion or harsh environments [51,52].

### 4.3. Analysis of Functional Genes of the Bacterial Community

The KEGG database contains a rich variety of functional genes that are responsible for almost all life activities of human beings and other animals, plants, and microorganisms on Earth. These functions amount to more than 40 kinds in the second level of the Kyoto Encyclopedia of Genes and Genomes (KEGG) metabolic pathways (Table S2). Similar to the functional genes responsible for the organic systems in the body, complex functional genes were found during different soil treatments [44]. However, the form in which these genes exist in the soil and their mechanism of action remains unclear.

In our study, we found that the functional genes detected in different sample types were consistent, and the types of functional genes present were the same. Through the analysis of these functional genes, we found genes responsible for basic metabolism, such as energy metabolism, amino acid metabolism, nucleotide metabolism, and carbohydrate metabolism. These functional genes are universal across all organisms and are called “housekeeping genes.” Housekeeping genes are ubiquitous in various living organisms, and they are highly conservative and are constitutive genes that are required to maintain the basic functions of cells [53].

## 5. Conclusions

In this study, we systematically studied the influence of different sample types, slope positions, and chemical properties of *C. camphora*-associated bacterial communities in different soils. Furthermore, we analyzed the changes in the predictive function of the bacterial community. Our study found that soil type is the main clustering factor of the *C. camphora* bacterial community and that root endosphere particularly enriched Firmicutes, relative to the other two sample types, while rhizosphere mainly enriched Acidobacteria. The bacterial alpha diversity of the *C. camphora* rhizosphere and bulk soil was higher in the downhill position, while the root endosphere was not affected by slope position. With regard to soil chemical properties, AN, TP, AP, AK, and TK had remarkable effects on the bacterial community. The predicted functional gene types in the different soil sample types of *C. camphora* were consistent.

The interactions of sample type, slope position, and microbial community of *C. camphora* are complicated, and their mechanism of action remains unclear. Therefore, further studies are necessary to explore how sample types and slope positions interactively drive the assembly of the *C. camphora* microbiome.

## Figures and Tables

**Table 1:** Mean relative abundances of top 10 differential phyla (%) in different sample types.

Phylum	Bulk Soil	Rhizosphere	Endosphere
Proteobacteria	29.88%	37.42%	25.59%
Acidobacteria	36.44%	35.42%	3.33%
Firmicutes	0.21%	0.28%	31.58%
Bacteroidetes	3.43%	3.87%	14.18%
Actinobacteria	5.45%	3.96%	7.45%
Chloroflexi	9.02%	4.17%	0.94%
Verrucomicrobia	5.37%	5.83%	2.03%
Cyanobacteria	0.08%	0.10%	9.28%
Planctomycetes	2.99%	3.26%	0.24%
WPS-2	3.25%	2.65%	0.17%
Others	3.87%	3.02%	5.21%

## Data Availability

The original sequences obtained by sequencing have been uploaded to the NCBI SRA database. The BioProject number is PRJNA779380.

## Supplementary Materials

The following are available online at https://www.mdpi.com/article/10.3390/microorganisms10010072/s1, Table S1: Soil chemical properties of different plots, Table S2: The corresponding pathways of the bacterial functions.
